# Systematic Review of the Literature to Inform the Development of a South African Dietary Polyphenol Composition Database

**DOI:** 10.3390/nu15112426

**Published:** 2023-05-23

**Authors:** Malory Jumat, Kwaku Gyebi Duodu, Averalda van Graan

**Affiliations:** 1 Biostatistics Research Unit, South African Food Data System (SAFOODS) Division, South African Medical Research Council, Francie van Zijl Drive, Parow Valley, Tygerberg 7505, Cape Town P.O. Box 19070, South Africa; averalda.vangraan2@mrc.ac.za; 2 Department of Consumer and Food Sciences, University of Pretoria, Private Bag X20, Hatfield 0028, South Africa; gyebi.duodu@up.ac.za; 3 Department of Global Health, Division of Human Nutrition, Faculty of Medicine and Health Sciences, Stellenbosch University, Francie van Zijl Drive, Tygerberg 7505, Cape Town P.O. Box 19063, South Africa

**Keywords:** polyphenol database, diet, health, systematic review, food composition database

## Abstract

Comprehensively compiled dietary polyphenol data is required to compare polyphenol content between foods, calculate polyphenol intake and study its association with health and disease. The purpose of this review was to identify data on the presence and content of polyphenolic components in South African foods, with the aim of compiling the data into a database. An electronic literature search was conducted up until January 2020 using multiple databases. Additional literature was sourced from South African university repositories. A total of 7051 potentially eligible references were identified, of which 384 met the inclusion criteria. These studies provided information on food item name, geographical distribution, polyphenol type, quantity, and quantification method. Data for 1070 foods were identified, amounting to 4994 polyphenols. Spectrophotometry was the main method used for quantification of gross phenolic content in various assays such as total phenolic content (Folin–Ciocalteu assay), total flavonoid content (AlCl_3_ assay) and condensed tannin content (vanillin–HCl assay). Phenolic acids and flavonoids were the main polyphenol classes identified. This review highlights that South Africa has abundant information on the polyphenol content of foods, which could be utilised within a food composition database for the estimation of polyphenol intake for South Africa.

## 1. Introduction

According to the World Health Organisation (WHO), non-communicable diseases (NCDs), such as cardiovascular diseases, cancer, diabetes and chronic respiratory diseases, are the leading global cause of death, accounting for 74% of deaths worldwide [1]. The most recently released Statistics South Africa (Stats SA) report on mortality and causes of death in South Africa shows that apart from tuberculosis (TB) as the main cause of death, diabetes mellitus was the second leading natural cause of death, followed by different forms of heart and cerebrovascular diseases [2]. Over the last 20 years, South Africa has experienced a significant nutritional transition, resulting in an increased intake of processed foods high in sugar, salt and saturated fats and an inadequate intake of fruit and vegetables [3]. A recent report found that the South African diet is unbalanced and consists mainly of meat and starch [4]. These dietary patterns have contributed to the high prevalence of obesity and other non-communicable diseases among the South African population [5]. A key message of the food-based dietary guidelines for South Africa [6] is to consume plenty of fruit and vegetables daily and there has been a multi-sector approach to promote the increased consumption of plant-based foods, especially those endemic to a specific area [4,5,7]. These approaches are deemed important as several epidemiological and intervention studies have reported positive correlations between plant food intake and the prevention of non-communicable diseases [8,9,10].

The positive correlation between plant food consumption and disease reduction has led the scientific community to identify specific compounds responsible for the observed health benefits. Scientific evidence linking increased consumption of plant foods to improved human health has been attributed in part to their content of beneficial polyphenols. Polyphenols are secondary plant metabolites that comprise a heterogeneous group of molecules that constitute a large family of more than 500 different compounds with highly diverse structures, from simple molecules, such as phenolic acids, to large ones, such as proanthocyanin polymers [11]. Polyphenols are common in many plant foods such as fruits, vegetables, cereal grains and beverages such as wine, beer and tea [12], which are food items consumed by many South Africans on a daily basis [13], while the most common phenolics in the human diet are phenolic acids, flavonoids and tannins [12]. During the last decade, interest in food polyphenols has increased substantially, especially among food scientists, nutritionists, the agricultural/food industry, and consumers. This is mainly due to the discovery of their antioxidant effects and their role in the prevention of non-communicable diseases [14]. Polyphenols are strong antioxidants that complement and add to the functions of antioxidant vitamins and enzymes as a defence against oxidative stress caused by excess reactive oxygen species [11,12]. The antioxidant action of polyphenols could potentially result in vasodilator, antithrombotic, anti-inflammatory, antiapoptotic, hypolipemic, or antiatherogenic effects that have been associated with decreased cardiovascular risk [15]. The antidiabetic effects of polyphenols are also multifactorial, as polyphenols have been shown to modulate the digestion of starch and other carbohydrates, induce satiety, mitigate non-enzymatic glycation, modulate hormonal responses and stimulate the secretion of glucagon-like peptide 1 (GLP1) [16]. Dietary polyphenols have also demonstrated anticancer activity, showing biological activity against the main cancer molecular targets such as kinases, pre- and anti-apoptotic proteins, enzymes that regulate energy metabolism and regulatory proteins linked to proliferation and signalling pathways. In addition, polyphenols have preventive effects against tumour initiation through numerous mechanisms, such as the avoidance of genotoxic molecule formation, the blockade of mutagenic transforming enzyme activity, the regulation of phase I and II enzymes, such as cytochrome P450s and S-transferase, as well as preventing DNA damage [17,18].

Furthermore, dietary polyphenol data can be used to compare polyphenol content between foods, to calculate polyphenol intake in populations and study its association with health and disease, or to evaluate the relative contribution of a given food product to the intake of a particular polyphenol as compared to other food sources [14]. Though there are international dietary polyphenol composition databases available for these purposes, research has shown that these databases lack variation and do not account for unique native foods as they are typically not region specific [19].

The content and composition of polyphenols in several South African fruits, vegetables, beverages, cereals and legumes have been documented in various publications. However, these publications are scattered and published in formats that are neither readily available nor formatted for the purpose of research and practice. Therefore, a need for a South African-specific polyphenol composition database that will provide accurate and validated composition data in a convenient, standardised and accessible format has been identified. The purpose of this review was to identify data on the presence and content of polyphenolic components in South African foods by means of a systematic review with the aim of compiling the data in a South African dietary polyphenol database as a supplement for the South African Food Data System (SAFOODS) [20].

## 2. Materials and Methods

A modified process based on the methodology used by Igwe et al. (2017) [19] and Probst, Guan and Kent (2018) [21] was employed to obtain South African dietary polyphenol data. The stages for this study consisted of (i) search strategy; (ii) eligibility screening; and (iii) data extraction and analysis. The structure of the review was conducted according to the Preferred Reporting Items for Systematic Review and Meta-Analyses (PRISMA) guidelines [22]. Though there are a limited number of reviews reporting on polyphenols found in specific foods, food groups, medicinal plants, and alcoholic and non-alcoholic beverages, to date, no systematic review has been done on South African polyphenol content as consumed in a regular diet from typical foods.

### 2.1. Search Strategy

A literature search of studies published (in the English language) was performed using Scopus, Science Direct, PubMed, Web of Science and ProQuest databases. A wide range of quantitative study designs were included if extractable data were available. Additional literature was sourced from South African university repositories. Web-based university repositories were used to search for relevant theses and dissertations. Search results were saved in Microsoft Excel © (Microsoft^®®^ Excel^®®^ for Microsoft 365 MSO, Version 2303) worksheets with hyperlinks to the abstract via the title. The theses/dissertations were independently screened via their abstracts. Discrepancies were resolved by consensus among the reviewers. The search terms and strategy used for the study selection were adjusted according to the database concerned but consisted of a combination of “South Africa” AND Food AND Diet AND drink AND Polyphenol OR flavonoids OR phytochemicals OR phenolic acid OR anthocyanidins OR isoflavones OR flavones OR flavonols OR flavanones OR hydroxycinnamic acid OR stilbenes.

### 2.2. Eligibility Screening

The studies included in this review had no date limitations as this was the first review of its kind and the aim was to obtain the maximum possible amount of data, though data was collected until January 2020. The dietary polyphenol database will be specifically focused on South African foods; therefore, data on foods not consumed in South Africa were excluded. Only studies published in English were considered for inclusion. All supplements, nutraceuticals, extracted and encapsulated forms of polyphenols, extracts from herbal/medicinal sources and purified or modified versions of polyphenols were excluded as the focus was on the inclusion of food items as consumed in a regular diet. Using a pre-defined eligibility criterion, the texts obtained via database searches were independently screened by their title and abstracts using the Rayyan web application in blind mode [23]. Theses/dissertations were also independently reviewed using an Excel spreadsheet with links to the abstracts of the theses/dissertations via the universities’ online repositories. Discrepancies between the two reviewers were resolved by re-evaluation of the specific texts until consensus was reached. If the data reported in an article and dissertations were identical, it was assumed that the source of the information was the same and therefore the information source with the least amount of information was excluded. The authors of the publications were contacted when necessary to obtain missing data or supplementary information.

### 2.3. Data Extraction and Analysis

The full texts of papers meeting the inclusion criteria were retrieved, further assessed for relevance, and summarised in tables. The following information was collected: Full publication reference, publication year, title, geographical location (province, town) of where the food item was obtained, food item name, food group, method of analysis/quantification, polyphenol class and polyphenol quantity. Discrepancies during the extraction phase were resolved by consensus of the reviewing team. The corresponding authors of papers were contacted when necessary to obtain missing or supplementary information. Data that were presented only in graph format were excluded from the final extraction. Data from the same food but from different publications were considered separately.

The extracted food items were grouped according to the SAFOODS food grouping system [15]. The total number of polyphenol values was calculated per individual food item as described using means with standard deviations, as reported in the original sources, when this information was available. Initial data aggregation was performed for the same food item with samples from various locations within the same province and/or analysed in different years and seasons. Polyphenols extracted from the included studies were grouped into polyphenol classes and subclasses according to Probst, Guan and Caldwell (2019) [21] and Phenol-Explorer [24]. The methodology used to group the polyphenol content extracted from the included studies was according to the classification used by Ignat, Volf and Popa (2011) [25]. Data collected were recorded in a Microsoft Excel© spreadsheet and basic analysis was performed using the Microsoft Excel Data Analysis Toolpak.

## 3. Results

### 3.1. Study Selection

**Figure 1:**
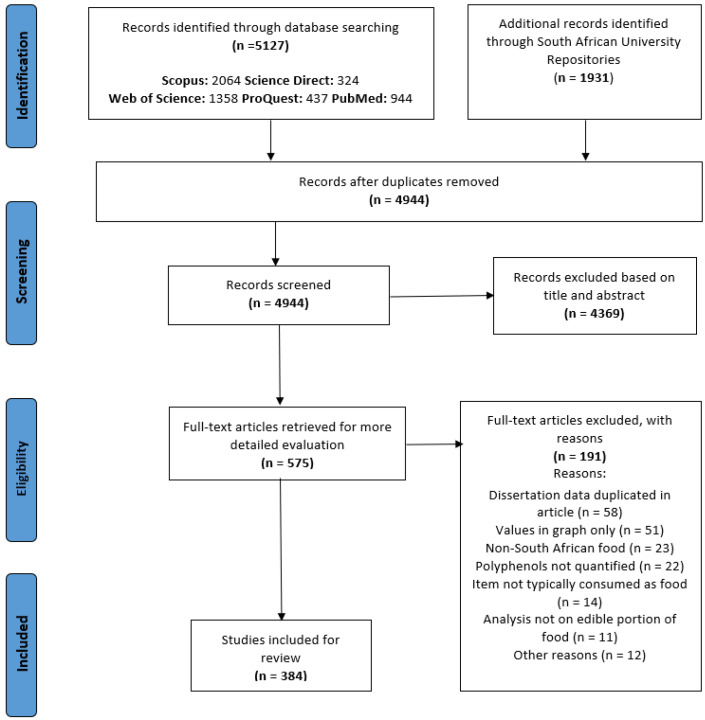
PRISMA flowchart of study selection process.

The search of the electronic databases identified 5127 potentially eligible articles. Additional records identified through South African university repositories identified 1931 potentially eligible master and doctoral dissertations/theses. After removing duplicates, 4944 records were screened. Subsequent to the initial screening based on title and abstract, 4369 records were excluded and 575 records remained for further full-text assessment. A total of 384 reports met the inclusion criteria and were ultimately included in the analysis. A PRISMA flow chart of the search strategy and selection process is depicted in Figure 1.

### 3.2. Year of Publication

**Figure 2:**
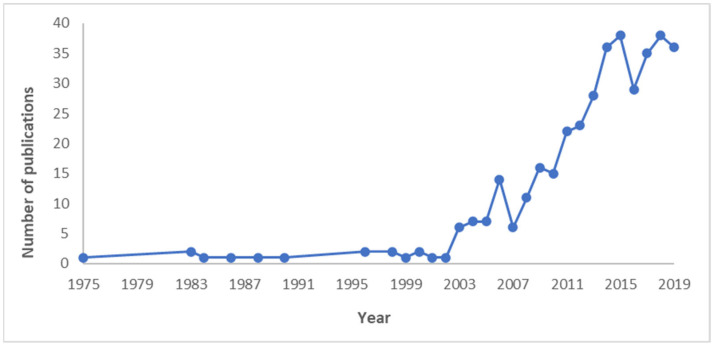
Number of publications per year in articles/theses/dissertations on polyphenols in South African dietary sources from over review period.

Figure 2 presents the distribution of included publications by year of publication. As shown, an upward trend in the number of publications was observed, with peaks in 2015 and 2018.

### 3.3. Data Sources

Scientific databases were the main sources of included publications (*n* = 218), while university repositories contributed 166 theses/dissertations. From the university repository data, Stellenbosch University was the main contributor of theses/dissertations, followed by the University of Pretoria and the University of KwaZulu-Natal (Table 1).

### 3.4. Geographical Distribution of Included Studies

**Figure 3:**
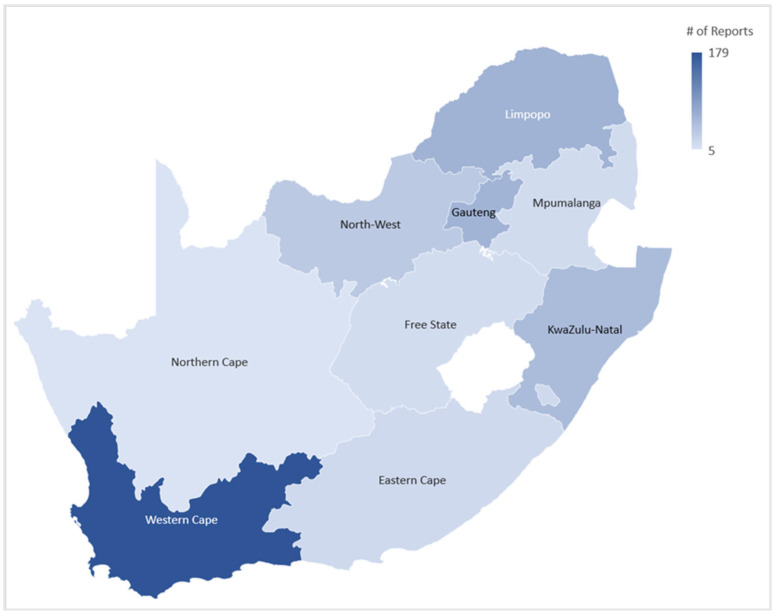
Geographical distribution of included publications (Author’s graphic, 2023).

An additional objective of the review was to identify the geographical location of the foods analysed in the studies. Thus, the city and province where food was sampled were extracted from the full text and calculated. If a publication sampled data from more than one province, the total number of provinces listed was added to the total. Figure 3 represents the provincial distribution of the studies.

### 3.5. Food Groups, Food Items and Total Polyphenols

The food items found in the publications fell into 12 of the SAFOODS food group categories, as shown in Table 2. The food group with the majority of food items and polyphenols is the miscellaneous group. This group contains food items such as wine and tea, which make up most of the food items extracted for this group. As expected, the meat and milk groups contained the least amount of food items and polyphenols. The legumes and legume products yielded the third highest amount of polyphenol data points, though they ranked only 5th highest in the total number of foods per food group.

### 3.6. Polyphenol Class and Subclass

**Figure 4:**
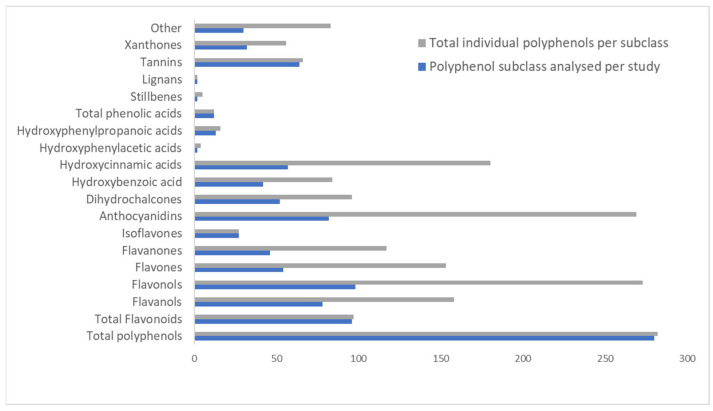
Distribution of polyphenol class and subclasses from included studies.

Figure 4 shows the distribution of polyphenol classes and subclasses from the included studies. Total polyphenols were reported in 73% of included studies (*n* = 280). Total flavonoids were reported in 96 studies. It must be noted that “total polyphenols” and “total flavonoids” referred to in this instance give only gross phenolic content and do not provide information about specific phenolic compounds. Looking at the flavonoid subclasses, flavonols and anthocyanidins were reported in 98 and 86 studies, both resulting in the identification of 273 polyphenols, respectively. Of the phenolic acids, hydroxycinnamic acids were identified in 57 studies, resulting in 180 polyphenols analysed at the study level, while some studies also reported on total phenolic acids (*n* = 12). The “other” polyphenol class was identified in 30 studies and resulted in 83 polyphenols. Only two studies reported on stilbenes and lignans, with 5 stilbenes and only 2 lignan types identified.

### 3.7. Polyphenol Quantification Methods

**Figure 5:**
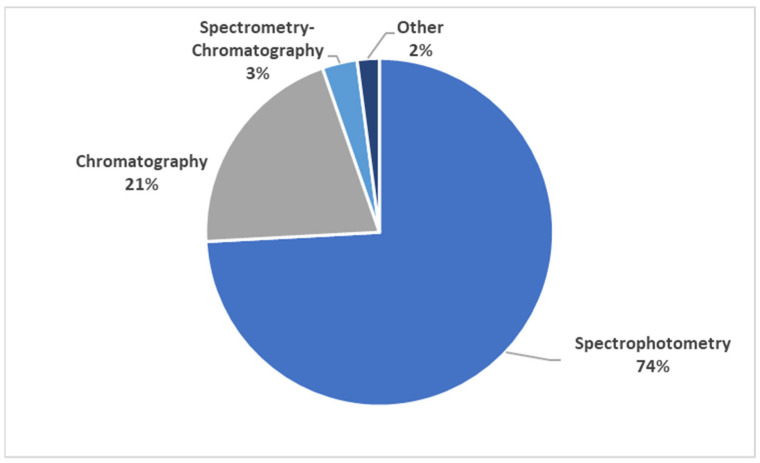
Distribution of methods used for quantification of polyphenols in included studies.

The main methods used for quantification of the extracted polyphenols are shown in Figure 5. Spectrophotometric methods were used by the majority of studies (74%) to quantify polyphenols followed by chromatography (21%). A combination of spectrometry and chromatography methods was used for analysis in approximately 3% of included studies. Spectrophotometric methods used to quantify gross polyphenol content include the Folin–Ciocalteu/Folin–Dennis method (total polyphenols), the vanillin/butanol–HCl method (tannin content), the aluminium chloride method (total flavonoids), pH differentiation (total anthocyanins), the 4-dimethylaminocinnamaldehyde (DMAC) method (proanthocyanidins) and the ferric ammonium citrate method (total polyphenols). Chromatographic techniques included high-performance liquid chromatography and gas chromatography. The majority of “other” techniques focused on precipitation-based methods.

## 4. Discussion

Food composition tables are an important and necessary tool for studying the relationships between diet and health, dietary intake assessments, nutrition labelling, policy development, as well as monitoring the quality of foods available to the consumer [26,27]. It is well recognised that adequate nutrition is a critical component of public health. Prior to the development and implementation of intervention programs to improve nutrition at the population level, it is important to first assess the nutritional status of a population [28]. Food composition databases are critical to converting food intake to nutrient intake to elucidate the relationship between food and specific dietary components consumed and their effect on various health outcomes. Over and underconsumption of certain dietary components could be indicative of the origin of certain health conditions. Furthermore, results from national health and nutrition surveys are used to inform food and nutrition policy in the form of food supplementation and fortification, dietary advice for pregnancy and the formulation of preventative diets for reducing diet-related non-communicable disease rates [29].

Although most food composition tables focus on energy and macro- and micronutrients, interest in non-nutritive components is increasing [28]. Polyphenols are of particular interest as their consumption is consistently linked with protection against non-communicable, chronic diseases [21]. This review showed numerous South African dietary polyphenol data available across multiple publications and study types.

Figure 2 demonstrates the increase in publications containing qualitative data on the number of polyphenols in food items over the last two decades. This shows the growing trend and, thereby, interest in studying and quantifying polyphenols in South African foods. This trend was also observed internationally across Europe, North America, South America, Asia and Australia [12,19,30,31,32].

It is known that knowledge syntheses such as systematic reviews are important to provide essential evidence to inform healthcare decision making [33]. Systematic reviews represent a rigorous approach to synthesizing and evaluating available scientific evidence. Findings from systematic reviews can be viewed as independent scientific publications to document the state of the scientific evidence, identify knowledge gaps and research needs, provide input into program and policy decision-making processes, and serve as the foundation for later updates as new data emerge [34]. Although systematic reviews are well known in the broader scientific/medical fraternity, their use in food composition is relatively new [19,21,35,36]. This systematic review was thus conducted due to the gap in knowledge of available polyphenol data and the need for such data in the country, as well as to provide scientific evidence to inform the South African Food Data System. It is also important to note that a critical component of a well-conducted systematic review is an objective, sensitive, and reproducible literature search of multiple sources, as was done in this review. Additionally, grey literature is deemed an important source of relevant information [37], as it may reduce publication bias, increase reviews’ comprehensiveness and timeliness, and foster a balanced picture of available evidence. Since failing to identify grey literature on a topic might affect the results of a systematic review. The repositories of all South African universities were used to search for grey literature, which yielded more detailed information in master’s and doctoral theses/dissertations. It is also standard practice to include theses/dissertations in the compilation of nutrient databases, as observed during the systematic update of the Argentinian food composition database, which included bachelor’s, master’s and doctoral theses [35].

An important objective of this review was to observe the geographical distribution of studies on dietary polyphenols throughout South Africa (Figure 3) and determine whether it follows the agricultural trends of the provinces. From the distribution of studies throughout the nine provinces of South Africa, the findings in this review support the notion that the distribution of studies on dietary polyphenols in South Africa mirrors the distribution of agriculture and food production trends in South Africa. According to the Census of Commercial Agriculture (CoCA) [38] in South Africa conducted in 2017, the Western Cape is the province that generates the most income from commercial agriculture, with the second highest number of commercial farms. The Western Cape is also the leading producer of grapes, apples, peaches, pears and plums and the number one producer of wheat, barley and onions. Furthermore, most of the included theses and dissertations originated from the University of Stellenbosch, while Cape Peninsula University of Technology contributed the 4th most included theses/dissertations (Table 1). Thus, it is expected that most data sources on the polyphenol content of food items would originate from the Western Province, as fruit and wine are large contributors of polyphenols to the human diet [31].

It was interesting that the second-largest number of studies originated from the Limpopo Province, although this province ranked 7th on the list of the number of commercial farms. However, according to the CoCA report, Limpopo is the leading producer of tomatoes and citrus fruits such as oranges, grapefruit, lemons, mangoes and tangerines [38]. Flavanones are a polyphenol subclass mostly associated with citrus fruits. Naringenin and hesperidin in particular are well-known polyphenols found in high volumes in the peel of citrus fruits [39]. Data from this review also found that the flavanone subclass was analysed in 46 of the included studies and yielded 117 individual polyphenols at the study level (Figure 4). Additionally, the Limpopo region is also the number one producer of avocado and butternut [38], which are both good sources of phenolic acids and flavonoids [40,41]. The Northern Cape was the province with the least number of included studies. Though it is the largest province in South Africa, it generates the least amount of income from commercial agriculture and the agricultural income generated is almost entirely centered around livestock [38]. Though there has been growing interest in the bioactivity of meats [32], they are not known sources of polyphenols.

Climate is an important driver of food production in South Africa, as the region is semi-arid [42]. Furthermore, rain is the main source of water for field crops in South Africa [38]; therefore, most agricultural production is focused on areas such as KwaZulu-Natal, Mpumalanga and Limpopo, where most rainfall occurs. The Northern Cape is the driest province in South Africa, with the lowest annual rainfall levels, indicative of the lowest percentage of agricultural activities, further explaining the reason for the least number of included studies [42]. Climate is also important for the production of wine, and the Mediterranean-like climate of the Western Cape is favoured in wine production, which yields the majority of wine produced in South Africa and thereby compares to the number of wine-related polyphenol studies seen in this review [43].

Besides the region-specific agricultural factors mentioned, population density is also a big driver for food production within a region and thus plays a role in the observed distribution of the included studies. According to the mid-year population estimates of 2022 conducted by Statistics South Africa [44], Gauteng comprises the largest contributor to the South African population, with approximately 16,10 million people (26.6%) living in this province. Though it is the densest province, it is also the province with the smallest agricultural surface. From this study, we observed that 62 studies were conducted in Gauteng, which ranked third after the Western Cape and Limpopo. Gauteng also houses four of the tertiary institutions from which theses and dissertation data were obtained (Table 1). KwaZulu-Natal is the province with the second-largest population, with an estimated 11.54 million people (19.0%) living in this province. KwaZulu-Natal Province houses three of the tertiary institutions from which repository data was obtained and fifty-three of the included studies were conducted in this province (Table 1; Figure 3). The Northern Cape remains the province with the smallest share of the South African population, and thus it was expected that the least amount of studies would originate from this province.

The two most widely used international polyphenol databases for the estimation of dietary polyphenol intake are the American USDA Database for the Flavonoid Content of Selected Foods [30] and the European Phenol-Explorer [24]. Although data quality assessment and data aggregation could lead to attrition in the number of food items finally included in the user database, this review found a preliminary number of 1070 food items that could potentially be included in the database, which is promising in light of the fact that the USDA Flavonoid Database and Phenol Explorer currently contain polyphenol data on 452 and 506 foods, respectively.

The distribution of foods according to the SAFOODS food groups follows the expected trend of consisting mainly of plant-based food items (>99%), as polyphenols are exclusive to plants. The miscellaneous food group, which contained the most food items (Table 2), mainly consists of wine and tea. Rooibos tea was investigated in the majority of studies involving tea. Rooibos tea is prepared from unfermented and fermented plant material from the Cape fynbos plant, *Aspalathus linearis,* and is a popular beverage among South Africans [45], while tea in general is one of the most frequently consumed foods [13]. Aspalathin and nothofagin, a xanthone subclass of polyphenols, are found in high concentrations in Rooibos tea, and many of the uniquely associated health benefits of Rooibos tea are attributed to these compounds [45,46]. Interestingly, the 2016 Tea Industry Landscape Report found that South African consumers are shifting from black tea consumption to Rooibos tea, most likely due to the health benefits associated with its consumption [47].

Wine is a well-known source of polyphenols, and thus it was no surprise that it accounts for a large number of food items extracted from the included studies. South Africa, in particular the Cape Winelands, is one of the largest producers of wine in the world, ranks eighth in overall volume production of wine and produces 4.1% of the world’s wine [43]. Therefore, research on the quality and quantity of polyphenols found in wine is of great importance for this industry. It was surprising to find so few articles analysing stilbenes (*n* = 2) since resveratrol is produced in grape skins and some of this solubilises into red wine during fermentation [48]. However, according to Vincenzi et al. (2013) [49], the concentration of resveratrol in red wine is too low to elicit a therapeutic effect, which could be the reason why there is not much research interest in quantifying it in wine.

Fruits were the third-largest food group and ranked second in terms of the number of polyphenols found within the group. South Africa is well known for its exportation of various citrus, apples and table grapes [38,50]. The country also has a rich diversity of indigenous fruit species that are often underutilised but have gained research interest due to their unique polyphenol content. A study conducted by Kucich and Wicht (2016) [51] compared, amongst others, the *Carissa macrocarpa* (num num), *Carpobrotus edulis* (sour fig), *Olea europaea* subsp. *Africana* (wild olive) and *Harpephyllum caffrum* (wild plum) fruits to commercial blueberries and cranberries. The sour fig, which is popular in the Western Cape, was found to have a total phenolic level comparable to cranberries. They also found that the wild plum, well known in the KwaZulu-Natal region, was found to have a high total polyphenol content, comparable to the total phenolic content of blueberries, which are popularly promoted as a “superfood” due to their bioactive components.

It was noteworthy that the third and fourth highest number of polyphenol data points were obtained from the legume and legume products group and the cereals and cereal products group, respectively. The legumes and legume products group accounted for 508 total polyphenols from only 75 food items (Table 2). This indicates that there is a particular interest in legume polyphenols in research conducted on South African foods. Legumes have been found to be an excellent source of polyphenols, including phenolic acids, flavonols, flavones, flavanols, flavanones, isoflavones, anthocyanins, tannins and other phenolics [52,53]. The Bambara groundnut and cowpeas make up the majority of food items within this group. Although the South African food-based dietary guidelines state that dry beans, split peas, lentils and soya should be consumed regularly [6], the Knorr Plate of the Nation report of 2020, which looks at the food consumption patterns of South Africans across various demographics, found that overall, South African meals contain minimal amounts (5 to 7%) of legumes [4]. However, with the global movement towards more plant-based diets, legumes have become important as a good source of plant protein [52]. Interestingly, in a recent review, Tian et al. (2019) [54] found that the content of polyphenols in grains is similar to that of fruits and vegetables and that some highly active phenolic compounds only exist in whole grains. Furthermore, the Knorr Plate of the Nation report also found that the current South African plate consists mostly of starches derived from refined grains [4]. The promotion of legumes and whole grain consumption could thus be an avenue to introduce more polyphenols into the South African diet.

A variety of polyphenol classes were extracted from the included studies. As fruit and vegetables were the food groups with the highest number of food items extracted, it was expected that high numbers of phenolic acids and flavonoids would be extracted, as was observed in Figure 4. Spectrophotometric methods provide useful qualitative and quantitative information and are the main technique used for the quantification of gross polyphenol content due to their simplicity and relatively low costs [25]. Thus, it is no surprise that spectrophotometric methods were the main methods used to quantify the polyphenol contents extracted in this study (Figure 5). However, it is known that spectrophotometric methods lack specificity for individual compounds and interference from non-polyphenol substances can cause false readings leading to overestimations [11]. High-performance liquid chromatography is a preferred method for the analysis of polyphenols in fruits due to the technique’s ability to separate compounds prior to analysis in order to accurately quantify and identify the individual polyphenols in foods [11,25]. With advances in the field of analytical chromatography, liquid chromatography-mass spectrometry has emerged as the go-to state-of-the-art analytical method for the identification and quantification of specific polyphenolic compounds in foods.

The main strength of the study is that the information obtained using the systematic process has delivered data that is diverse across the country, and the foods analysed varied according to the agricultural characteristics of each region. Inclusion of the university repository search in the strategy has provided numerous data that would not have been identified if only scientific databases were searched. Furthermore, upon completion of the user database, the possibility of an enhanced scope of improved study results is possible for South African dietary polyphenol intake studies. Some limitations include the exclusion of date restrictions when conducting the search and thus some data that met the inclusion criteria were analysed using methods that are currently not considered good quality. The Folin–Ciocalteu method has been critiqued as it lacks specificity for phenolic compounds and is known to overestimate values when other reducing agents are also present in the foods, compared to the more specific high-performance liquid chromatography (HPLC) methods [55]. Nonetheless, as this was the first stage in the process leading to the development of a national polyphenol database and in the absence of any prior knowledge of the extent of data available, it was deemed important to obtain as much data as possible.

Future studies should include the assessment of data quality through adaptations of internationally available tools such as EUROFIR’s Quality Index guidelines [56] and the USDA’s data quality evaluation system [57,58] to the South African-specific data. Additionally, the obtained data should be compiled into a reference database to enable comparisons and estimations of dietary intake. Moreover, a process of data validation of the compiled data is envisioned to further strengthen the quality of the compiled data.

## 5. Conclusions

In this study, dietary polyphenol data from South African foods were systematically searched for using various sources. This review highlights that South Africa has numerous sources of data on the polyphenol content of foods. Furthermore, it has shown that it could be utilised within a food composition database for the estimation of dietary polyphenol intake in the South African population.

## Figures and Tables

**Table 1:** University included reports distribution.

University	Theses/Dissertations Included
Stellenbosch University	59
University of Pretoria	31
University of KwaZulu Natal	18
Cape Peninsula University of Technology	17
Tshwane University of Technology	7
University of Limpopo	6
Durban University of Technology	5
North-West University	4
University of Johannesburg	4
University of the Western Cape	4
University of South Africa	3
University of Zululand	3
University of Venda	2
Central University of Technology	1
University of Cape Town	1
University of Fort Hare	1

**Table 2:** Distribution of food items and polyphenols from included publications per food group.

Food Group	Number of Food Items	Number of Polyphenols
Miscellaneous	383	2482
Vegetables	187	458
Fruit	163	839
Cereal and cereal products	145	432
Legumes and legume products	75	508
Beverages	74	213
Soups, sauces, seasonings and flavourings	17	32
Fats and oils	11	11
Nuts and seeds	10	12
Sugar, syrups and sweets	3	5
Meat and meat products	1	1
Milks and milk products	1	1
**Total**	**1070**	**4994**

## Data Availability

Data presented in this study are available on request from the corresponding author.
